# Curative multistage embolization of symptomatic multiple (supra- and subtentorial) arteriovenous malformations with a distal flow-related aneurysm

**DOI:** 10.1093/jscr/rjag636

**Published:** 2026-07-28

**Authors:** Andrey Petrov, Azamat Nazarbekov, Arkady Ivanov, Anna Petrova, Konstantin Samochernykh, Ruslan Sharshebaev, Larisa Rozhchenko

**Affiliations:** Vascular Neurosurgery Department, Polenov Neurosurgical Research Institute, Branch of Almazov National Medical Research Centre, 191014 Saint Petersburg, Russia; Vascular Neurosurgery Department, Polenov Neurosurgical Research Institute, Branch of Almazov National Medical Research Centre, 191014 Saint Petersburg, Russia; Vascular Neurosurgery Department, Polenov Neurosurgical Research Institute, Branch of Almazov National Medical Research Centre, 191014 Saint Petersburg, Russia; Vascular Neurosurgery Department, Polenov Neurosurgical Research Institute, Branch of Almazov National Medical Research Centre, 191014 Saint Petersburg, Russia; Vascular Neurosurgery Department, Polenov Neurosurgical Research Institute, Branch of Almazov National Medical Research Centre, 191014 Saint Petersburg, Russia; Vascular Neurosurgery Department, Polenov Neurosurgical Research Institute, Branch of Almazov National Medical Research Centre, 191014 Saint Petersburg, Russia; Vascular Neurosurgery Department, Polenov Neurosurgical Research Institute, Branch of Almazov National Medical Research Centre, 191014 Saint Petersburg, Russia

**Keywords:** arteriovenous malformations, flow-related aneurysm, embolization

## Abstract

We present a unique case report of curative three-stage embolization of multiple bilateral cerebral arteriovenous malformations of supra- and subtentorial localization (left temporal lobe, vermis and right cerebellar hemisphere) combined with a flow-related aneurysm of the P4-P5 segments of the right posterior inferior cerebellar artery. A patient in their 40s. Clinical manifestation in 2011 (over 10 years ago) with intracerebral haemorrhage. During three stages of embolization with non-adhesive embolic materials (Squid 12,18, BALT), all arteriovenous malformation (AVMs) were obliterated and the flow-related aneurysm was reduced. Both transarterial and transvenous approaches were used. Adenosine-induced cardioplegia and pressure cooking (in the case of transvenous approach) were used to reduce flow and control reflux. This example shows that the combination of modern technological methods and an optimal strategy of sequential endovascular embolization with non-adhesive compositions can be an effective and safe method for the treatment of multiple AVMs, leading to favorable clinical outcomes.

## Introduction

Brain arteriovenous malformation (AVM) is a relatively rare vascular pathology with an incidence of 1.1–1.4 cases per 100 000 population per year [1]. According to the definition of Pilipenko *et al.* (2020), multiple AVMs of the brain are two or more well-defined tangles of pathological vessels with (according to angiography) different feeding arteries and different drainage veins [2]. According to various literature sources, multiple AVMs occur in 0.15%–4% of patients with AVMs [1–3]. Since its first description by Zellem *et al.* (1985) [4] and up to now in the English language literature we have found a description of 39 cases of multiple cerebral AVMs [2, 3, 5]. The combination of supra- and subtentorial multiple AVMs was found in six (15.4% of 39) cases [2, 3, 5, 6]. The incidence of aneurysms combined with AVMs varies from 2.3% to 58% in different studies [7]. The incidence of bleeding from multiple AVMs is 71.4% [2]. No cases of total endovascular embolization of multiple AVMs have been described. We present a unique observation of radical three-stage embolization of multiple cerebral AVMs combined with a flow aneurysm.

## Case report

**Figure 1:**
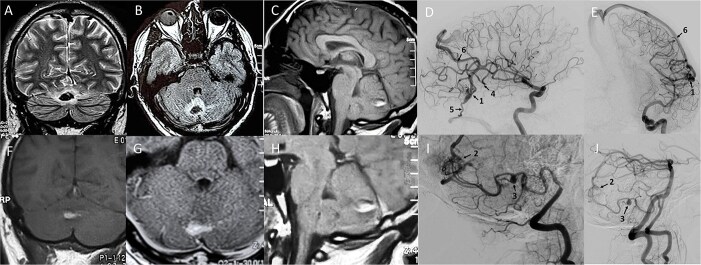
Preoperative neuroimaging. A, B, C, F, G, H - MRI of the patient’s brain in his 38, showing cerebellar vermis hematoma. D, E, I, J - digital subtraction angiography (DSA) following left internal carotid artery (D, E) and right vertebral artery injections (I, J). A - coronal T2 weighted MRI; B - axial T1 weighted MRI; C - sagittal T1 weighted MRI without contrast; D –DSA of left ICA lateral view; E - DSA of left ICA frontal view; F - coronal T1 weighted MRI with contrast; G - axial T1 weighted MRI with contrast; H - sagittal T1 weighted MRI with contrast; I - DSA of right VA lateral view; J - DSA of right VA lateral view. Black arrows (on D, E, I, J): 1 - arteriovenous malformation (AVM) of the left temporal lobe, Spetzler-Martin II; 2 - cerebellar AVM, Spetzler-Martin II; 3 - flow-related aneurysm on the afferent from the right PICA; 4 - afferent from the left middle cerebral artery; 5 - an efferent draining into the vein of Labbe; 6 - an efferent draining into the vein of Trolard.

The adult patient was admitted to a neurosurgical hospital with complaints on headaches and unsteadiness when walking. It was known from anamnesis that ~10 years ago the subject had an acute intracranial hemorrhage in the cerebellar worm. The patient received conservative treatment with good effect. MR angiography of the brain revealed multiple bilateral cerebral arteriovenous malformations of supra- and subtentorial localization (left temporal lobe, vermis and right cerebellar hemisphere) and a fusiform aneurysm of the distal part of the right posterior inferior cerebellar artery (PICA) (Fig. 1).

We decided to perform the separate, staged embolization of two AVMs. Due to the fact of previous hemorrhage, the subtentorial malformation was regarded as having a higher risk of rupture and was chosen as the first stage of treatment.

**Figure 2:**
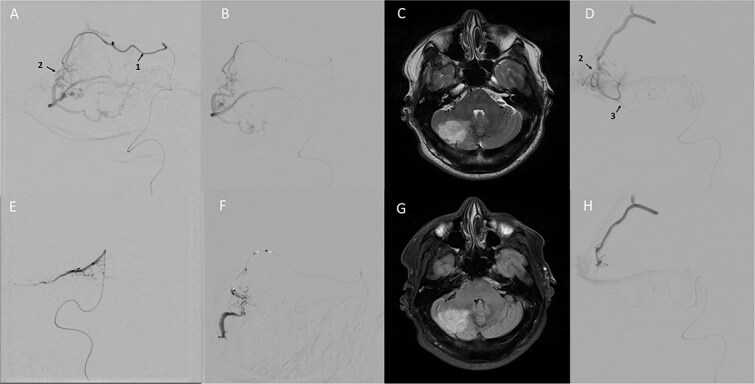
First and second stages of cerebellar AVM embolization. A, B - DSA in lateral view shows contrasting of the subtentorial AVM from the afferent of the right superior cerebellar artery; E,F - first stage of embolization in frontal (E) and lateral (F) views, the images show the spread of the embolic agent - Squid 12,18 (Balt); C,G - postoperative brain MRI (C- axial T2 weighted MRI, G - axial T1 weighted MRI) showing the area of cerebellar ischaemia developed after the first stage of embolization; D - DSA in lateral view showing contrasting AVM from the afferent of the right PICA; H - second stage of total embolization of AVMs from the afferent of the right PICA, the image shows the spread of the embolic agent - Squid 18 (Balt). Black arrows indicate: 1 - afferent from superior cerebellar artery; 2 - nidus of cerebellar arteriovenous malformation; 3 - afferent from the right PICA.

**Stage 1**: transarterial endovascular partial embolization of the worm and the right cerebellum hemisphere AVM through the right superior cerebellar artery with non–adhesive composition SQUID 12 - 1 ml. In the postoperative period there was a transient neurological deficit - dysgraphia and dysarthria (regression within 1.5 months), which developed as a result of postembolization ischemia of the cerebellum (Fig. 2). mRs −1.

**Stage 2:** in 7 months: Total transarterial embolization of the worm and the right cerebellum hemisphere AVM through the PICA using Squid 18–2.5 ml (Fig. 2). No neurological deficit was observed. mRs 0.

**Figure 3:**
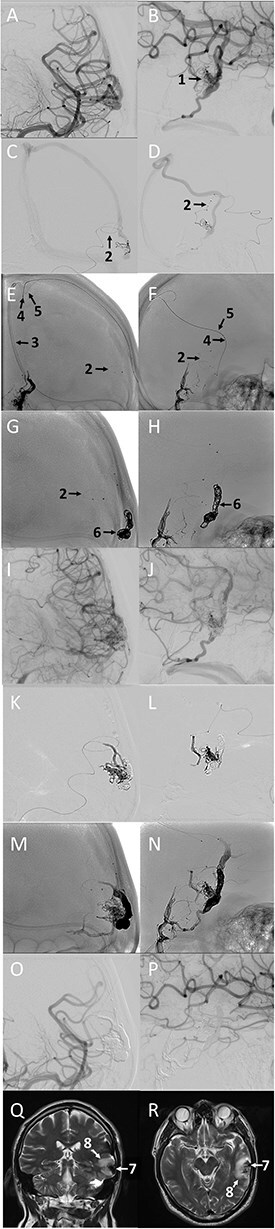
Third stage - total embolization of left temporal lobe AVM from combined transarterial and transvenous approach. A, B - DSA from the left ICA in frontal (A) and lateral (B) views; C, D - DSA from the left middle cerebral artery afferent, frontal (C) and lateral (D) views; E,F - craniograms showing the tools placement before embolization, frontal (E) and lateral (F) views; G, H - craniograms showing the complex of coils in the venous drainage of AVM, frontal (G) and lateral (H) views; I, J - DSA from the left ICA showing AVM contrasting after positioning of coils in the venous drainage, frontal (I) and lateral (J) views; K,L - transarterial embolization with non-adhesive embolic agent - Squid 18 (Balt), frontal (K) and lateral (L) views; M, N - craniograms showing carst of non-adhesive composition and coil’s complex after transvenous stage of AVM embolization with embolic agent - Squid 18 (Balt), transvenous ‘pressure coocking’ technique, frontal (M) and lateral (N) views; O, P - DSA from left ICA after total embolization, frontal (O) and lateral (P) views; Q, R - postoperative brain MRI showing postoperative edema around the total embolized AVM of the left temporal lobe, coronal T2 weighted (Q) and axial T2 weighted MRI (R) views. Arrows: 1 - nidus of the left temporal lobe AVM; 2 - afferent from the left middle cerebral artery; 3 – Guide catheter in the superior sagittal sinus; 4 - sonic 1.5 F 45 (Balt) microcatheter placed in the AVM draining vein; 5 - headway 17 microcatheter (Microvention) positioned in the draining vein; 6 - coil complex placed in the AVM draining vein; 7 - embolized AVM of the left temporal lobe; 8 - perifocal edema around the total embolized AVM.

**Stage 3:** 4 months later: Combined transarterial-transvenous embolization of AVM of the left temporal lobe (Spetzler-Martin II) using SQUID18–4.5 ml. A flow arrest technique - adenosine-induced cardioplegia (13 s)- was used (Fig. 3). A control angiography revealed no filling of the subtentorial AVM. According to our internal protocol, embolization was performed under general anesthesia, and then in the neuro-intensive care unit medical sedation was extended for 20 hours. After control magnetic resonance imaging (MRI) scans and digital subtraction angiography (DSA), the patient was awakened without neurological deficit. mRs 0.

**Figure 4:**
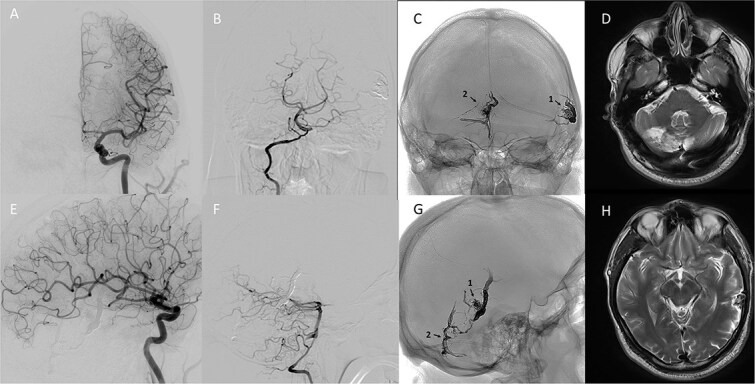
3 months follow up. A, E - DSA from the left ICA showing absence of cerebral AVM contrast, frontal (A) and lateral (E) views; B, F - DSA from the right vertebral artery showing absence of cerebral AVM contrast, frontal (B) and lateral (F) views; C, G - craniograms showing embolic agent’s carsts of multiple cerebral AVMs, frontal (C) and lateral (G) views; D - T2 weighted brain MRI in axial view showing formed postischemic cerebellar cyst; H - T2 weighted brain MRI in axial view showing regression of edema around a total embolized AVM of the left temporal lobe. Arrows: 1 - Squid (Balt) carst in the left temporal lobe AVM; 2 - Squid (Balt) carst in the cerebellar AVM.

The brain MRI on the second day revealed signs of dimethyl sulfoxide (DMSO)-associated vasogenic edema around the embolized AVM. A 1 month later brain MRI revealed the increased area of vasogenic edema, which regressed in 3 months. On the control DSA, multiple AVM were totally excluded from the blood flow, and the AVM-associated aneurysm was reduced (Fig. 4). mRs 0.

## Discussion

**Figure 5:**
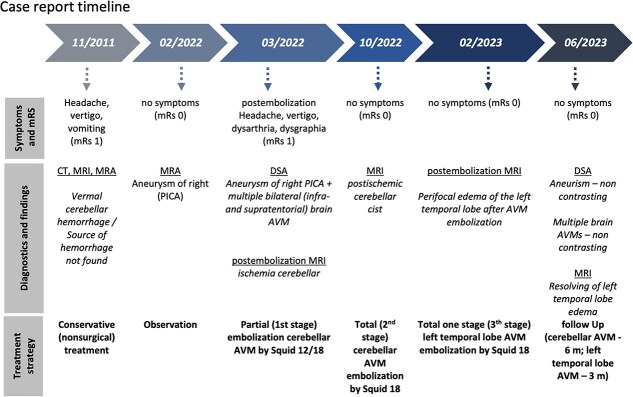
Case report timeline.

Among published cases only six publications described a combination of supra- and subtentorial localization [3, 5]. Three of them had hemorrhage due to one of the AVMs ruptures, in the fourth case a sourse of haemorrage was a flow-related aneurysm. It is known that among posterior fossa AVMs, flow-related aneurysms are found in 13% of cases [7]. In our case, the AVMs were localized bilaterally sub-(right) and supratentorially (left), there was a flow-related aneurysm of the right PICA. According to the shape of the haemorrhage, it could be assumed that the source of the subtentorial haemorrhage was cerebellar AVM. When choosing treatment tactics, most authors were guided by the presence and source of haemorrhage. Their tactics ranged from observation [6] to active surgical interventions [3, 5]. The only radically treated case with multiple supra- and subtentorial AVMs was the case presented by Tada *et al.* (1986) [5]. The disease manifested by haemorrhage from supratentorial malformations, and the patient underwent two microsurgical operations within 1.5 months. In a publication of Robert *et al.* (2016) [3], in three cases of supra- and subtentorial multiple AVMs, partial embolization of haemorrhage sources was performed. In our case, treatment was also initiated with embolization of a ruptured subtentorial AVM with associated aneurysm, which had a high risk of re-rupture. The modern techniques (adenosine-induced cardioplegia, the use of transvenous access in combination with transarterial), the advantages of non-adhesive compositions of different viscosities Squid 12,18 (Balt) [8], allows to perform radical endovascular treatment of AVMs of various complexity, including multiple ones (Fig. 5).

## Conclusion

We presented a unique clinical case of a previously undescribed radical endovascular treatment of multiple bilateral supra- and subtentoral AVMs. This example indicates that a combination of modern technological techniques and an optimal strategy for sequential endovascular embolization with non-adhesive compositions can be an effective and safe method of treating complex multiple AVMs, leading to favorable clinical results.

## Data Availability

No additional data are available beyond the information presented in the manuscript due to patient confidentiality.
